# The Relationship of Palliative Care With Assisted Dying Where Assisted Dying is Lawful: A Systematic Scoping Review of the Literature

**DOI:** 10.1016/j.jpainsymman.2019.12.361

**Published:** 2020-06

**Authors:** Sheri Mila Gerson, Gitte H. Koksvik, Naomi Richards, Lars Johan Materstvedt, David Clark

**Affiliations:** a School of Interdisciplinary Studies, Dumfries Campus, University of Glasgow, Scotland, United Kingdom; b Faculty of Humanities, Department of Philosophy and Religious Studies, Norwegian University of Science and Technology (NTNU), Trondheim, Norway

**Keywords:** Assisted suicide, euthanasia, assisted dying, palliative care, hospice

## Abstract

**Context:**

A central approach of palliative care has been to provide holistic care for people who are dying, terminally ill, or facing life-limiting illnesses while neither hastening nor postponing death. Assisted dying laws allow eligible individuals to receive medically administered or self-administered medication from a health provider to end their life. The implementation of these laws in a growing number of jurisdictions therefore poses certain challenges for palliative care.

**Objectives:**

To analyze the research literature about the relationship of assisted dying with palliative care, in countries where it is lawful.

**Methods:**

A five-stage scoping review process was adapted from the Joanna Briggs Institute. Data sources searched through October 2018 were MEDLINE, CINAHL, PsychINFO, SCOPUS, and ProQuest dissertations and theses, with additional material identified through hand searching. Research studies of any design were included, but editorials or opinion articles were excluded.

**Results:**

After reviewing 5778 references from searches, 105 were subject to full-text review. About 16 studies were included: from Belgium (*n* = 4), Canada (*n* = 1), Switzerland (*n* = 2), and the U.S. (*n* = 9). We found that the relationship between assisted dying and palliative care practices in these locations took varied and sometimes combined forms: supportive, neutral, coexisting, not mutually exclusive, integrated, synergistic, cooperative, collaborative, opposed, ambivalent, and conflicted.

**Conclusion:**

The studies in this review cast only partial light on challenges faced by palliative care when assisted dying is legal. There is pressing need for more research on the involvement of palliative care in the developing practices of assisted dying, across a growing number of jurisdictions.

## Introduction

A central aspect of the World Health Organization definition of palliative care is that it neither hastens nor postpones death.^1^ Assisted dying, as it gains momentum in laws around the world, therefore creates challenges for the practice of palliative care.^2^^,^^3^ Most palliative care associations oppose assisted dying and are often vocal in their opposition.^4^, ^5^, ^6^ In some instances, however, there is evidence of divided opinions, for example, within the Association of Palliative Medicine in the U.K.^7^ One view is that these professional associations are there to promote palliative care and not to oppose assisted dying.^8^ Some palliative care organizations take a stance by issuing declarations about assisted dying and seek to influence public opinion and policy makers in the process.^2^^,^^3^ A study of 104 palliative care and assisted dying declarations from around the world showed that palliative care declarations did not *define* assisted dying, but most campaigned against it. Conversely, some declarations saw assisted dying alongside or even as a part of palliative care.^2^

There is no one agreed definition of assisted dying, but for the purposes of this review, we use the umbrella term assisted dying to encompass euthanasia, physician-assisted suicide, and assisted suicide,^9^^,^^10^ whereby an individual can lawfully receive medically administered or self-administered medication from a health provider to end their life at their own competent and voluntary request.

There is also no one agreed on definition for palliative care, although that of the World Health Organization from 2002 still has widespread currency and describes palliative care as an approach and a philosophy that improves the quality of life of patients with life-limiting illnesses and families through addressing psychological and spiritual needs and include some notion of holistic care delivered through a multidisciplinary team.^11^ Palliative care encompasses end-of-life care and includes hospice care, but how this care is provided depends on cultural and geographical settings.^12^

Research on assisted dying practices in Canada, the U.S., and some European countries indicates that 74%–88% of persons who opt for assisted dying also receive hospice or palliative care services.^13^ Assisted dying and palliative care practices have each developed separately in differing periods and geographies, making it difficult to understand how they can and do relate to each other. Countries with established palliative care systems *and* assisted dying laws that have been implemented for more than 10 years include jurisdictions within the U.S.,^14^^,^^15^ Belgium,^16^ Luxembourg,^17^ and The Netherlands.^18^

Systematic reviews have explored several aspects of the relationship between assisted dying and palliative care, including attitudes and experiences of professionals toward euthanasia or assisted suicide;^19^, ^20^, ^21^ the desire for or the wish to hasten death and how palliative care practitioners may respond to it;^22^, ^23^, ^24^, ^25^ and information about the practice of euthanasia or assisted suicide in different jurisdictions.^26^ To date, however, there is no literature review that specifically addresses if or how assisted dying is integrated within or rejected by palliative care practices, once it is made lawful.

### Research Question

To identify associations between palliative care and assisted dying, our research questions had to be broad rather than closely prescribed. We therefore agreed not to define any particular categories of relationship between the two in the research question. The research questions were as follows:

• What does the research literature reveal about the relationship between assisted dying and palliative care in contexts where assisted dying is lawful?
• What can be learned from the selected studies to inform future research and practice?

## Methods

We conducted a systematic scoping review. This method was chosen because of the broad nature of the research questions and a need to include disparate forms of evidence in the review, avoiding judgments about research quality. Scoping reviews are useful tools to identify research gaps, examine emerging evidence, and identify what remains to be investigated, but with a rigorous and transparent process.^27^^,^^28^ We were guided in our review by the methodology outlined by the Joanna Briggs Institute and further developed by Khalil et al.^29^ This method involves a five-stage review process: 1) identifying the research question, 2) identifying relevant studies, 3) study selection, 4) presenting data in a tabular and narrative format, and 5) collating the results to identify implications of findings for policy, practice, or research. Before embarking on the present review, therefore, we developed a study protocol that defined the objectives, methods, and proposed plan and gave consideration to matters of good practice.^29^ The protocol helped to identify terms that described both assisted dying and palliative care, and the relationship between them, which were subsequently used as search terms in the review.

### Identifying Relevant Studies

Five databases were searched by S.M.G. and G.K. between September and October 2018: MEDLINE , CINAHL, PsychINFO, SCOPUS, and ProQuest Dissertations and Theses. The three-step approach identified by Khalil et al.^29^ suggests first completing an initial search of MEDLINE and CINAHL before adapting and varying the terms to widen the scope of the search (see the Appendix for an example of the initial search strategy applied to MEDLINE and CINAHL). This initial search was completed in consultation with a reference librarian. Search terms were separated by category and are identified in Table 1. We conducted a full-text search of the articles for terms related to palliative care, hospice, and assisted dying. Additional terms, such as terminal care, palliative or terminal sedation, were excluded to focus only on studies that mention and include palliative care, hospice, and assisted dying. The text was also searched for terms related to the possible relationship between assisted dying and palliative care, for example, rejection, collaboration, and integration. The second step of the approach involved applying search terms to all databases.

**Table 1:** Search Terms

Search terms associated with assisted dying, assisted suicide, physician-assisted suicide, and euthanasia	“Assisted dying” OR “assisted suicide” OR Euthanasia NOT Animals OR “voluntary euthanasia” OR “aid in dying” OR “physician assisted dying” OR “physician aid in dying” OR “physician assisted suicide” OR “medical aid in dying” OR “medical assistance in dying” OR “Death with Dignity”
Search terms associated with palliative care	“Palliative care” OR Hospice* OR “Palliative care nursing” OR “palliative medicine”
Search terms associated with relationship	Integrat* OR Relation* OR Compatib* OR Consequence* OR Rejection* OR Collaboration* OR Cooperat* OR impact* OR impede* OR embed* OR oppose* OR improve* OR involve* OR harm*

### Study Selection: Inclusion and Exclusion Criteria

Table 2 lists our final inclusion and exclusion criteria. First, publications that were exclusively opinions, personal views, or perspectives about assisted dying and palliative care were excluded in favor of publications based on enquiry and investigation. Second, studies that did not include data from after implementation of the assisted dying law in the given jurisdiction were also excluded. Third, studies that did not specifically involve palliative care or hospice were excluded, for example, studies that investigated nurses or general practitioners but which had no identified focus on palliative care.

**Table 2:** Inclusion and Exclusion Criteria

Category	Inclusion	Exclusion
Type of sources	Research studies using any methodology published in English	Opinions, perspectives, views, and editorials Research from before implementation of laws/court ruling Research from areas that do not have lawful assisted dying
Setting	Palliative care inpatient, outpatient, hospice, home-based hospice or palliative care	Articles that do not include hospice or palliative care
Population	Adult, pediatric	
Intervention	Assisted suicide or euthanasia and palliative care	

### Presenting the Data

**Fig. 1:**
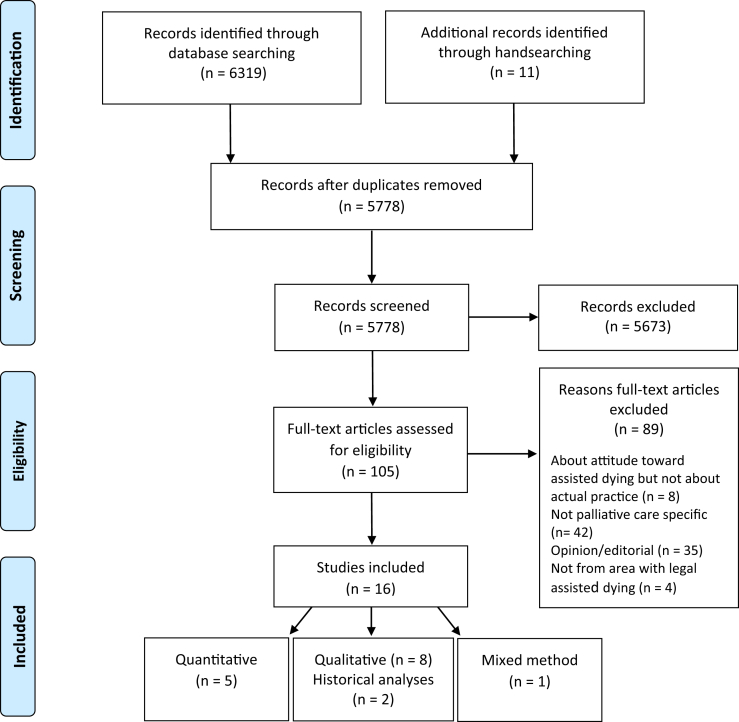
Preferred Reporting Items for Systematic Reviews and Meta-Analyses flowchart illustrating the search strategy. *From*: Moher D, Liberati A, Tetzlaff J, Altman DG, The PRISMA Group (2009). Preferred Reporting Items for Systematic Reviews and Meta-Analyses: the PRISMA statement. *PLoS Med* 6:e1000097.

The flowchart in Fig. 1 details the process of the search and the final results of study selection.

The search resulted in 5778 citations after duplicates were deleted. The third step of the literature search included analysis of the reference lists of the final identified articles. Reference lists were hand searched and identified 11 additional studies that met the criteria for review. Titles and abstracts were reviewed against these criteria by S. M. G. and G. K., who then reviewed 105 full-text articles and in collaboration with all team members, agreed on the final 16 articles for inclusion.^30^, ^31^, ^32^, ^33^, ^34^, ^35^, ^36^, ^37^, ^38^, ^39^, ^40^, ^41^, ^42^, ^43^, ^44^, ^45^ To present the data, a spreadsheet was created with column headings denoting study design, date and location of data collection, population and setting studied, key findings, and explicit or implied relationship of assisted dying to palliative care. Findings from the studies were then extracted from the results, discussions, and conclusions. The selected studies covered a range of research methods: two retrospective mortality studies;^34^^,^^44^ two postal surveys;^33^^,^^40^ a chart review;^45^ three documentary analyses of assisted dying policies in palliative care institutions;^31^^,^^32^^,^^41^ four qualitative interview studies;^35^^,^^36^^,^^42^^,^^44^ one focus group study;^38^ one mixed method survey;^40^ one review of the rise of palliative care and assisted dying in one location;^39^ and one historical analysis that included empirical data and interviews.^30^

Table 3 lists the characteristics and components of each study, its design, and results. The column data collection date indicates that the study data were collected after the implementation of the relevant assisted dying law for the jurisdiction. The last column specifies the relationship between palliative care and assisted dying practices. The results and discussion section of each article were examined to understand how the relationship was described in the study. A quote or an example from each relevant study is included in this last column to emphasize the description of the relationship. Most included studies identify explicitly how specific palliative care professionals relate to or work with assisted dying laws and local policies within their practice. Other included studies have relationships that are implicit in the data and may describe more than one type of association.

**Table 3:** Characteristics/Components of Included Studies (*n* = 16)

Article	Study Design	Data Collection Year(s)	Location	Population and Setting	Key Findings	Relationship of AD to Palliative Care (Explicit or Implied)
Bernheim et al.^39^	Historical analysis based on epidemiological studies and regulatory guidelines	Before 2008	Belgium	Nurses and physicians trained in both palliative care and euthanasia—home and hospital setting	Advocates for legalization of euthanasia were palliative care clinicians. The development of palliative care and the process of legalization of euthanasia can be mutually reinforcing. The process of legalization of euthanasia was ethically, professionally, politically, and financially linked to the development of palliative care	Explicit: integral, synergistic Integral palliative care … euthanasia is considered as another option at the end of a palliative care pathway and the patient's preferences come first
Bernheim et al.^30^	Historical analysis of integral model follow-up from 2008 study	Before 2013	Belgium	History of debate with all relevant groups	Relationship between palliative care and AD is described as synergistic and integral Palliative care and euthanasia can exist separately when the patient does not want palliative care or it can go hand in hand with each other	Explicit: no antagonism, integral, integrated, and embedded Integral palliative care is thus conventional palliative care with an *integrated* possible choice of the option of euthanasia
Bittel et al.^40^	Questionnaire sent to 90 physicians, 286 nurses, and 28 other association members	2000	Switzerland	Physician members of Swiss Association of Palliative Care	Members of Swiss Palliative Care Association have varying relationships with right-to-die organizations: Physicians prescribe lethal medications (8%) Willing to assist a patient to die (40%) Members opposed (56%)	Implied: opposed, ambivalent, and cooperative Personal experiences with AD revealed that a minority have participated although it is against the bylaws of the association
Campbell & Black^31^	Analyses and summary of 30 policy documents from 33 of 35 hospice programs	2012	Washington State, U.S.	Professionals working with patients receiving home hospice services	Values in AD policies: relief of pain and suffering, information disclosure, respect refusal, compassionate care, nonabandonment, enhance quality of life, respect patient choice, respect patient-physician, refrain from hastened dying 78% of hospice programs restrict staff presence when patients ingest lethal medications	Implied relationship: opposed, ambivalent, cooperative, and complementary *Models of participation*: Opposition (21%)—restricted from participating in patients request Nonparticipation (33%)—staff are not allowed to participate in process (18%) or action (15%) Noninterference (21%)—a matter between patient and physician and not responsibility of hospice Respect patient choice (24%)—accepts responsibility to make sure patient has information and access—hospice and AD complementary
Campbell & Cox^41^	Analyses and summary of 40 policy documents about AD from 86% of state-affiliated hospice programs	2009	Oregon, U.S.	Professionals working with patients receiving home hospice services	Reports actual practice may not reflect policy Positions of 55 hospice programs on AD: • Hospice incompatible with assisted death (4%) • Noncooperation (4%) • Opposition (9%) • No direct participation (11%) • Follow statutory provisions (16%) • No active participation (18%) • Respect self-determination (18%) • Nonparticipation (20%)	Implied relationship: ambivalent, cooperative, opposed, and conflicted *Models of participation*: Full participation (16%)—staff provide information about AD, may refer to physician, and permits staff to be present with patient and family at the time AD medication is ingested Moderate participation (32%)—staff may provide information about AD and may be present when patient takes lethal medications if requested Limited participation (27%)—staff may refer to physician for information but the presence not permitted when patient ingests lethal medications Nonparticipation/noncooperative (25%)—no referral or participation in any way with AD
Campbell & Cox^32^	Analysis of policy or educational documents about AD from 56 of 65 programs (includes data from 2010 article)	2009–2010	Oregon	Professionals working with patients receiving home hospice services	The documents revealed a diversity of hospice values on AD: • Respect for patient self-determination • Neither prolong nor hasten death • Respect physician-patient relationship • Enhance quality at end of life • Nonabandonment • Compassion • Dignity • Sacredness of life	Implied relationship: overall, ambivalent, but evidence of collaborative, cooperative Generally, hospice programs assume a minor role in decision-making process and set boundaries around six key caregiving considerations; language, collaboration with doctors, provision of lethal medications, assistance with taking the medications, and staff presence at death
Carlson et al.^33^	Quantitative—postal survey 50 of 77 hospice chaplains	2003	Oregon	Chaplains working with hospice patients, primarily home based	Chaplains help patients explore the relationship between religious and spiritual beliefs and AD More than half of respondents have worked with patients who chose AD and do not feel they influence decisions although only 40% said they supported the law	Implied relationship: cooperative, ambivalent Chaplain's “deliver support to patients no matter what the patient's final decision” regarding AD
Dierickx et al.^34^	Population-based mortality follow-back study—random sample of 687 deaths	2013	Belgium	Involvement of palliative care with patients who requested euthanasia	Palliative care professionals were involved in decision making and performance of euthanasia in nearly 60% of deaths by euthanasia. Patients requesting euthanasia more likely to have palliative care. Palliative care is offered to every patient who requests euthanasia, but there are some who do not wish for it	Explicit and implied: embedded, not contradictory Euthanasia and palliative care do not seem to be contradictory practices
Gamondi et al.^35^	Qualitative interviews with 23 physicians	January–February 2015	Switzerland	Palliative care physicians working with patients	Palliative care physicians' role in assisted suicide is not clearly defined. One-third of physicians consider AD as a tool in palliative care, one-third ambivalent, and one-third strongly opposed, saying no place for assisted suicide in palliative care	Explicit and implied: opposition, ambivalent, and conflicted Palliative care physicians can be actually acting in isolation and secrecy when confronted with AD requests resulting potentially in ethical dilemmas, and possible collateral damage
Gerson^36^	Qualitative interviews—seven nurses, seven social workers, three chaplains, and three physicians	2015	Washington State, U.S.	Home hospice professionals	Professionals are confused about policy but work with patients even when they do not agree with their choice for AD. Indicates relationship varies depending on professional group and interpretation of hospice institutional policy	Explicit and implied: tension, challenged, not mutually exclusive There is a relationship between hospice and AD containing tension and challenges … but that home hospice and AD are not mutually exclusive
Harvath et al.^42^	Qualitative interviews—20 nurses, hospice social workers	After implementation of law. Year unspecified	Oregon, U.S.	Home hospice professionals	Dilemmas exist around whether AD is antithetical to hospice care and whether their employer permits them to give information about, or work with patients choosing AD	Explicit and implied: evidence of collaboration, conflicted, antithetical opposed Hospice professionals may experience conflict between wanting patients to have transformational experiences or support family wanting to care for them as long as possible, and supporting patients' desire to be in control of death
Miller et al.^37^	Quantitative—postal survey of 306 nurses and 85 social workers	2001	Oregon, U.S.	Home hospice nurses and social workers	Hospice social workers generally more supportive of AD than nurses. About 95% of all surveyed report that hospices should be either supportive or remain neutral	Explicit and implied: supportive, neutral, not mutually exclusive Most hospice professionals in Oregon do not believe that assisted suicide and hospice enrolment are mutually exclusive alternatives
Miller et al.^43^	Qualitative pilot project—exploring experiences of the three authors who are social workers	After implementation of law. Year unspecified	Oregon, U.S.	Social workers in health systems, outpatient, and acute care settings	Some concerns identified that the Death with Dignity Act is at odds with hospice philosophy, especially in religious institutions. Dilemmas arise as some professionals feel satisfaction that they are able to accompany the patient and family, while others feel complicit or negligent. Social workers with longer experience in hospice are more comfortable with AD	Implied: conflicted, cooperative Social workers in religiously based health care systems were faced initially with what felt like conflicting mandates of health system not to discuss AD, which conflicted with. Profession's code of ethics aiming for patient self-determination, but understanding was reached allowing them to satisfy both directives
Norton and Miller^38^	Qualitative—focus group with nine hospice social workers	After implementation of law. Year unspecified	Oregon, U.S.	Hospice social workers primarily home based	There is a lack of clear and consistent policy in for social workers to follow. Social workers weigh the values of hospice and their code of ethics and their role is not clear cut	Implied: conflicted, ambivalent Ambivalence within team can impact patients' access … Policies conflicted with patient or employee values creating dilemmas for decision making
Van den Block et al.^44^	Quantitative—retrospective mortality study—surveyed end-of-life decisions among 1690 nonsudden deaths in 181 (2005) and 174 (2006) practices	2005–2006	Belgium	Nonsudden deaths and euthanasia and other end-of-life decisions in last three months of life	AD and other end-of-life decisions are not related to a lower use of palliative care. AD occurs with multidisciplinary care. Receiving spiritual care is associated with higher frequencies of AD than receiving little spiritual care	Explicit and implied: coexist, synergistic Life shortening and end-of-life decisions often occur within the context of multidisciplinary care and coexist with a palliative care philosophy
Wales et al.^45^	Quantitative—retroactive chart review of 45 patients in a home palliative care setting assessed for MAiD	June 17, 2016–June 30, 2017	Toronto, Canada	Patients receiving home palliative care	Results suggest that MAiD can be successfully integrated in home-based palliative care with emphasis on collaboration; however, challenges persist related to serving large geographic area, medication delivery, and well-being of community partners. Differences in perspectives among MAiD providers and those who are conscientious objectors	Explicit: integrated, collaborative… emphasis on collaboration … accommodating different comfort levels with MAiD among small group of providers

AD = assisted dying; MAiD = Medical Assistance in Dying.

### Collating the Results

The fifth step in the chosen review process is collating the results, summarizing, and reporting the findings. To do this, we developed a preliminary inductive synthesis of the findings, which involved a combination of hand coding and coding using NVivo 12 software (by S. M. G. and G. K.). We created a coding framework that specified the jurisdiction and the type of relationship that was described in the content of each article. S. M. G. and G. K. also itemized terms describing the relationship between assisted dying and palliative care. We undertook an inductive thematic analysis, following Braun and Clarke,^46^ and subsequently categorized the results by country and context.

## Country and Context

### Belgium: Integral and Synergistic

Assisted dying became legal in Belgium in 2002. The law is not limited to those who are terminally ill, and a patient can request self-administered or medically administered medications from a physician to end their life.^16^

The four studies from Belgium^30^^,^^34^^,^^39^^,^^44^ present palliative care and assisted dying coexisting in a largely unproblematic and even synergistic way. They defend the integral palliative care model, which is seen in stark contrast to an antagonistic relationship that exists in other jurisdictions.^39^ For example, Bernheim et al.^39^ found that in Belgium in 2008, palliative care and assisted dying were not mutually exclusive and were inextricably linked in their development: The process of legalisation of euthanasia was ethically, professionally, politically, and financially linked to the development of palliative care.^39^ The study by Bernheim et al.^30^ in 2014 responds to critiques of the 2008 analysis. Most of the opposition or challenge to the 2008 article, the authors report, was from international critics who expressed a range of ideological and moral concerns rather than raising issues in relation to practical implementation. The authors claim that there are unique aspects related to the culture of Belgium that allow assisted dying to be included as one element within an array of health care options. This includes a tradition of liberalism and secular humanism … at all levels of society.^30^ Unlike other jurisdictions, legislation permitting assisted dying in Belgium was initiated by palliative care physicians in a context where palliative care is considered an accessible health care option for all.^47^

In two of the included articles, Bernheim et al.^30^^,^^39^ explained the origins and functioning of the Belgian system. These articles are included in the review because they report on empirical data collected after the euthanasia law was passed and because they report explicitly on the relationship between palliative care and euthanasia and how this has emerged in Belgium. Integrated end-of-life care offers the option of euthanasia, and a palliative care team is involved in most cases of euthanasia in the country. The authors imply that palliative care professionals have expertise in conducting sensitive conversations about death and dying, which helps with assessing and working with individuals who request assisted dying. Therefore, general physicians associated with the Life-End Information Forum also undergo palliative care training provided by the Palliative Care Federation of Flanders. Importantly, palliative care does neither have a monopoly on the provision of assisted dying nor are palliative care physicians legally required to participate in the assessment or practice of it. Conscientious objectors must, however, disclose and justify their stance in the early stages of caring for a patient. The studies do not indicate the incidence or prevalence of conscientious objection among palliative care professionals.

A mortality-based retrospective study of deaths in the region of Flanders, Belgium, by Van den Block et al.^44^ explored the presence of palliative care services in the final three months of life. The researchers found that assisted dying was more prevalent in inpatient palliative care units than in hospitals or care homes. The study also suggests a strong relation between the provision of spiritual care (as part of palliative care practice) and opting for an assisted death. The authors suggest that this might be because provision of spiritual care helps patients to express their wishes, including desire for an assisted death, or that spiritual or existential care is more likely to be provided in response to such a request. Dierickx et al.^34^ in their population-based study also reported that 71% of individuals who requested euthanasia in Belgium were involved with palliative care services and that palliative care professionals were involved in decision making and or performance of euthanasia in 60% of cases.

In the Belgian context, the terms integral and synergistic are used by palliative care providers to describe the relationship (or model) of palliative care and assisted dying. Integral palliative care is described and defined as conventional palliative care that offers the option of euthanasia.^30^ Synergistic refers to the combined development and promotion of palliative care and euthanasia.^39^

### Toronto, Canada: Integrated and Collaborative

Medical Assistance in Dying (MAiD) was made lawful in the Canadian province of Québec in December 2015 and legalized in the country as a whole in June 2016.^48^ This may account for only one article meeting the inclusion criteria. With the exception of Québec that only allows euthanasia by a physician, the law in Canada allows clinician-administered or self-administered medications for a patient with a grievous and irremediable medical condition.^49^ Clinician-administered euthanasia entails that nurse practitioners may also inject a patient with lethal medications, making the Canadian situation unique in the world as no other jurisdiction allows nonphysicians to perform euthanasia. To be eligible, a patient must be suffering from a serious incurable illness but does not require a specified time-limited prognosis.

A retrospective chart review by Wales et al.^45^ examines a process for implementing MAiD in home-based palliative care settings in Toronto. Within a team of physicians, some had agreed to take part in all aspects of MAiD, others to undertake MAiD assessments but not administer the lethal medications, whereas others would not agree to take part in any aspect of MAiD. An institutional process was developed to accommodate the different stances of the physicians while making sure that all patients requesting MAiD were assessed and, if eligible, received medicalized assistance. The process relied on conscientious objectors adhering to regulations to transfer MAiD responsibilities to another clinician. The authors conclude that MAiD can be successfully integrated into home-based palliative care and that diverging opinions within the medical community on the topic can be acknowledged and accommodated within the institution's internal processes.

The study suggests a relationship that is integrated and collaborative because the institution's palliative care policy seeks to find alternatives for a patient who requests MAiD but whose physician conscientiously objects to being involved. The study, however, does not discuss how, or indeed if, palliative care team members other than physicians may have been involved in the assisted dying requests or administering of MAiD.

### Switzerland: Ambivalent, Cooperation, and Opposed

Assisted suicide carried out by laypersons has been legally condoned in Switzerland's penal code since 1946. However, laws regulating assisted suicide in nursing homes and hospitals have been passed in the Cantons of Vaud and Neuchatel.^35^^,^^50^ A doctor must examine the patient and write the lethal prescription but is not present at the death. In most cases, the assisted suicide is administered by various right-to-die organizations that operate according to their own rules.^51^

Despite Switzerland's long history of decriminalized assisted suicide, only two studies were identified that met the review criteria. Bittel et al.^40^ outlined findings from a survey conducted in 2000 by the Swiss Association for Palliative Medicine, Care and Support about members' opinions and positions on physician-assisted suicide and euthanasia. In relation to the aims of our review, members were also asked about their personal experiences with the practice. Eight percent of physicians reported that they had practiced assisted suicide, despite this practice being against the bylaws of this Swiss Palliative Care Association. Forty percent of physicians and nurses said that they would be willing to assist a patient to die, but 56% said that they were opposed to assisted suicide and 69% said that they were opposed to direct active euthanasia—which in The Netherlands and Belgium is called euthanasia only, without either of the prefixes used in the Swiss study.

Gamondi et al.^35^ conducted an interview study in 2015 with 23 palliative care physicians in Switzerland exploring perspectives on, and involvement in, assisted suicide. Those interviewed said they regularly received requests for assisted suicide, but none had had any official training in how to deal with such requests, and they rarely acted on the patient's request or supported patient access to assisted suicide. A third of the interviewed physicians considered assisted suicide a tool in palliative care, a third were strongly opposed to assisted suicide, and a third were ambivalent. Most participants saw it as the responsibility of the patient, rather than the doctor, to make contact with right-to-die organizations to facilitate an assisted suicide but did not see it as their role to intervene in the process or prevent contact. Overall, it was rare for a physician to advise a patient to make contact with a right-to-die organization. Gamondi et al.^35^ concluded that the Swiss model gives palliative care physicians opportunities to develop roles that are compatible with their own values, whether they correspond to the expectations of patients. They suggest that specific education for all palliative care professionals and more structured ways to manage communication concerning assisted suicide are warranted.

The two included studies from Switzerland demonstrate that physicians do not actively participate in offering assisted suicide as part of palliative care. The term ambivalent participation therefore describes their orientation, where participation is influenced by their own personal values. The term cooperative is also included because there are some physicians in Switzerland whose specific education, training, and belief system are conducive to being favorable to assisted dying requests. However, the term opposed is also included because both studies included here indicated that at least a third of participants were opposed to assisted suicide. The studies only narrowly describe practical experiences of palliative care providers with patients who seek an assisted suicide, and details of actual practice are absent.

### Oregon and Washington, U.S.: Cooperative, Conflicted, Not Mutually Exclusive, and Opposed

Several jurisdictions in the U.S. have passed laws allowing physicians to prescribe a dose of lethal medications for an eligible patient to self-administer.^14^^,^^15^^,^^52^, ^53^, ^54^, ^55^, ^56^, ^57^ As of July 2019, jurisdictions included California, Colorado, District of Colombia, Hawaii, Maine, New Jersey, Oregon, Vermont, and Washington. The practice is also lawful in the state of Montana after a court ruling in 2019.^58^ In all states, health care providers, including physicians, are not required to be present at the patient's death. To be eligible, an individual must have a six-month life-limited prognosis and be deemed mentally competent.^59^

Nine of the included studies are from the U.S., seven from the state of Oregon,^32^^,^^33^^,^^37^^,^^38^^,^^41^, ^42^, ^43^ and two from the state of Washington.^31^^,^^36^

Three of the studies are specifically about hospice institutional policies on assisted dying. These studies suggest that although some hospice programs have policies that allow staff to cooperate with patients who request assisted dying, other institutions have less categorical policies, and this can cause dilemmas among staff about how to practice.^31^^,^^32^^,^^41^ These studies make the point that policy may not reflect practice, especially if practitioners are not provided with requisite training about the law. In a 2010 study about hospice programs, nearly half had policies that suggested moderate to full participation with assisted dying, with only 25% that did not officially participate in what they called physician-assisted dying.^41^ The study by Campbell and Cox,^32^^,^^41^ however, only addresses policy and lacks information about the actual experiences of palliative care practitioners when they are with patients who are requesting, and making use of, assisted dying legislation. As the authors state, “what is stated in policy and what happens in practice can be two quite different things.^41^”

Miller et al.^37^ noted that after legislation was passed in 1997, Oregon hospices had no policies either on assisted dying or that opposed it. In their survey of 306 hospice nurses and 85 social workers, they found that 62% had discussed assisted dying with at least one patient in the past year. They reported that 95% of all those surveyed believed that hospices should take either a neutral stance or support patients' requests and allow assisted dying as a part of hospice care.

Norton and Miller^38^ conducted a focus group study with nine hospice social workers from different hospice programs across the state of Oregon. The study revealed a lack of clear and consistent policy for social workers to follow, leaving them unclear about their role, albeit supportive of assisted dying as an option for their patients. The interview study of Harvath et al.^42^ with 20 hospice nurses and social workers similarly concluded that there are dilemmas around whether assisted dying is antithetical to hospice care and confusion about the legal and professional boundaries in terms of what constitutes assistance. For some of the nurses and social workers interviewed, discussing assisted dying with patients was viewed as an opportunity to discuss fears and concerns about dying and potentially bring more attention to symptom control. For others, lawful assisted dying introduced new professional dilemmas including an increased sense of responsibility to alleviate all symptoms or to convince patients to make another choice. However, despite challenges, participants in the study worked with patients who sought assisted dying.

The survey by Carlson et al.^33^ of 50 hospice chaplains in the state of Oregon discovered a range of views about the Death with Dignity Act and chaplains' involvement with the act. Although those surveyed were divided in their views with almost equal numbers supporting and opposing the act, none stated that they had refused to minister to a patient who chose assisted dying. Half of respondents had worked with a patient who had made an explicit request to make use of the act. The study does not include many details about the frequency or content of interactions between the chaplains and patients who desired an assisted death. The authors conclude that most chaplains surveyed understood their role as primarily providing a nonjudgmental listening presence—an approach that suggests a cooperative relationship between hospice chaplain services and assisted dying.

The interview study by Gerson^36^ examines the experiences of hospice professionals in Washington state and suggests that many institutions officially prohibit professionals from either providing information to patients about assisted dying or being present when an individual ingests the lethal medications. Nurses, social workers, and chaplains reported that they consented to patients' wishes to be present when the lethal medications were ingested even if they believed the policy of their employer prohibited their involvement. These findings support the results of the study by Campbell and Black^31^ who collected documents from 33 of 35 hospice programs in Washington. They report that nearly 78% (*n* = 26) of the hospice programs have a policy that prohibits hospice staff from being present either when a patient ingests the lethal medications or between ingestion and death. The authors reported that 21% (*n* = 7) of programs are opposed to assisted dying—staff being prohibited from participating in a patient's request; 33% (*n* = 11) are nonparticipating but through a commitment to nonabandonment they will refer patients; 21% (*n* = 7) have policies of noninterference—whereby hospice staff are instructed not to influence the patient's choice and to leave the decision between the physician and the patient, a relationship that is deemed to be outside the domain of hospice care; and 24% (*n* = 8) support patient choice and give their patients full information about their legal right to an assisted death.^31^

The terms cooperative and not mutually exclusive are used to describe the relationship of palliative care with assisted dying here because included studies indicate that many palliative care professionals and institutions cooperate with patients' requests. Still others may choose to be present with patients at the time of the planned death, even when the law does not require a professional to be there. The terms conflicted and opposed are also used because the existing evidence indicates that assisted dying is not integrated into palliative care practice, is not without dilemmas, and may depend on individual values that may not concur with organizational or professional policies.

## Discussion

Our first research question asked what the research literature reveals about the relationship between assisted dying and palliative care in contexts where assisted dying has been made lawful. We categorize the relationship in the four countries where there was relevant literature as variously: supportive, neutral, coexisting, not mutually exclusive, integrated, synergistic, cooperative, collaborative, opposed, ambivalent, and conflicted. The evidence about this relationship, however, is limited from the studies reviewed here. It therefore remains unclear how palliative care practitioners have responded to such legalization, and how practice has been influenced by related institutional or professional policies.

The small number of studies that met our inclusion criteria suggests that there are those who work in palliative care who either offer assisted dying themselves or who cooperate with patients requesting assisted dying by referring to participating physicians or organizations. At the same time, it seems that palliative care professionals are often unclear about their role in assisted dying. What is lacking in the Belgian studies is detailed and nuanced insight into how euthanasia is introduced within a palliative care context and whether and how this varies within and across institutions or professional groups. Indeed, none of the selected articles explain how the synergistic relationship takes form in practice.

The studies from the U.S., Switzerland, and Canada paint a more complex picture of shifting relationships between assisted dying and palliative care. Whether the findings of these studies may be extended to the situation in all Switzerland, Canada, or in all U.S. states that allow assisted dying, we do not know, although it would seem unlikely. First, many palliative care physicians in Canada reportedly object to the MAiD law,^60^ and second, there are indications that neither palliative care nor assisted dying is entirely accessible.^61^^,^^62^ The shifting relationship between palliative care and assisted dying in any jurisdiction may not be the same as in other jurisdictions, even within the same country.

The studies from Switzerland illustrate a situation where a lack of formal training in communication relating to assisted dying, along with an absence of clear guidelines, leave palliative care physicians to act in accordance with their own individualized ethical frameworks. Moreover, the situation suggests that there is little consistency for patients if physicians do not have a protocol to follow. As with Switzerland, the research from Oregon and Washington confirms the need for clearer policies and guidelines to support palliative care professionals who are often unclear about their role in assisted dying.

Our second research question asked what can be learned from the selected studies to inform future research and practice. Only articles from Belgium, Oregon, and Washington specifically identified how palliative care organizations or policy relate to assisted dying. In addition, most of the articles from the U.S. were about the state of Oregon, despite assisted dying laws being implemented in other areas during a period of two to 10 years (California, Colorado, District of Columbia, Montana, Vermont, and Washington). The absence of research from these specific U.S. states is a notable gap that requires attention, together with the international dearth of relevant studies that we have identified, most obviously from The Netherlands.

## Limitations

This review explicitly sought out publications based on enquiry and investigation to shed light on the different relationships that exist between palliative care and assisted dying once assisted dying is lawful. We were looking specifically for articles addressing a relationship between the two interventions and used terms to define them. We chose to exclude editorials, opinions, and perspective pieces that are not subject to the same validity checks as research studies, and which, while contributing to wider debate, may lack an empirical evidence base. However, a future scoping review *could* be undertaken, which included such pieces to see how the results would compare to those presented in this study.

## Conclusion

The studies in this review cast only partial light on the challenges faced by palliative care at the level of policies, guidelines, and individual practices when assisted dying is legal. Where evidence does exist, subtle dilemmas, uncertainties, and variable actions emerge, except in Belgium where the two extant studies show the opposite. Belgium is the only jurisdiction where the medical specialty of palliative care has developed in tandem with assisted dying, resulting in the integral model of palliative care, particularly in the region of Flanders where data were collected for one of the selected studies.^44^ The Belgian situation is therefore unique. Whereas elsewhere the legalization of assisted dying has been met with general reticence if not opposition from many medical and palliative care associations,^2^^,^^5^ this was not the case in Belgium where to the contrary, the Federatie Palliatieve Zorg Vlaanderen^63^ supports assisted dying, and palliative care physicians were instrumental in advocating for and developing practices to enable it.^30^^,^^39^ Despite this synergy, there is still a shortage of detailed empirical studies from Belgium about how palliative care is delivered in tandem with assisted dying and whether, for example, there are variations in practice depending on the institution or the patient's medical condition.

The authors of the studies from Belgium were the only ones clearly in support of assisted dying. Studies from Switzerland, Canada, and the U.S. did not have explicit conclusions in support of or in opposition to assisted dying legislation. Although there is a vast amount of commentary, even polemic, before legalization, on the likely effects on palliative care,^64^, ^65^, ^66^, ^67^, ^68^ there appears to be very little research on the impact of assisted dying on palliative care once legislation is introduced. This might be because opposition is often based on principled beliefs, which do not require evidence of their veracity, namely that assisted dying is morally wrong,^69^ or it might be that assisted dying is not wrong but should be kept separate from palliative care practices.

There was no research from The Netherlands that matched the criteria for inclusion in this review. The search revealed several studies about the experiences of euthanasia from the perspective of general practitioners,^70^, ^71^, ^72^, ^73^ but none that were specific to palliative care and assisted dying. An ethnographic study examining euthanasia discourse in The Netherlands among general practitioners, patients, and families indicated that talk about euthanasia has become another form of palliative care.^74^ Norwood^74^ suggested that allowing, encouraging, and supporting people to talk about what is important to them at the end of life is indeed a palliative measure, even when it is about the desire for euthanasia. In addition, an analysis by Gordijn and Janssens^75^ described the history and development of palliative care and euthanasia in The Netherlands, but the experiences reported are from data collected before the implementation of the 2002 law. Overall, in the Dutch context, palliative care interventions appear to have been integrated within secondary care and general practice,^74^^,^^76^ but the relationship between assisted dying and palliative care *specifically* is not discussed in these studies.

Likewise, we found no research from Luxembourg that met our inclusion criteria. Government documents report that Luxembourg is in line with the Belgian model and that palliative care has developed in collaboration with euthanasia and assisted suicide, but there is no research evidence to corroborate these statements or provide any details about what such integration looks like in practice in this particular country context.^17^^,^^77^

Our review therefore raises many questions. How does a patient-centered multidisciplinary palliative care team work with patients who have voiced an interest in opting for an assisted death? What happens when palliative care teams want to support a patient in their decision to choose an assisted death but then come into conflict with institutional policy or practice that rejects assisted dying as an option? What happens if evidence suggests palliative care does work in conjunction with assisted dying once legislation has shifted the boundaries of what is permissible? And how, in a practical sense, does a synergistic approach work for a patient who is receiving palliative care and then opts for euthanasia, or alternatively, how might those seeking euthanasia then receive palliative care? There is a need for more in-depth understanding of how palliative care practices interact with the implementation of assisted dying in different cultural and legal contexts. This is a rapidly evolving field, and it is imperative that there is up-to-date research into how palliative care is responding and the impacts of legislation on the specialism.

There is a strong likelihood that more laws will be passed granting people the possibility of an assisted death in different jurisdictions around the world. For example, the Death with Dignity Act was signed into law in the U.S. state of Maine in June 2019, and the New York legislature is actively considering the Medical Aid in Dying Act.^58^ Also, in June 2019, the Australian state of Victoria legalized both euthanasia and physician-assisted suicide.^78^ The debate on legalizing assisted dying in the U.K. continues with a recent poll of the Royal College of Physicians switching from an oppositional to a neutral stance.^79^ In Germany, assisted dying advocates have challenged a 2015 law that outlaws commercial-assisted suicide.^80^ More research is needed about the different types of involvement of palliative care practitioners—and not just physicians—in the developing practices around assisted dying. This not only has consequences for the holistic care of people at the end of life but also for the overall discipline and philosophy of palliative care.

## Figures and Tables

**Table:** 

S33	S12 AND S17 AND S32	1214
S32	S18 OR S19 OR S20 OR S21 OR S22 OR S23 OR S24 OR S25 OR S26 OR S27 OR S28 OR S29 OR S30 OR S31	7,455,759
S31	TX harm*	178,550
S30	TX involve*	1,740,648
S29	TX improve*	1,995,267
S28	TX oppose*	49,593
S27	TX embed*	115,842
S26	TX impe*	245,650
S25	TX impact*	913,982
S24	TX cooperation*	101,118
S23	TX collaboration*	81,747
S22	TX rejection*	104,034
S21	TX consequenc*	373,883
S20	TX compatib*	104,190
S19	TX relation*	2,436,366
S18	TX integrat*	583,024
S17	S13 OR S14 OR S15 OR S16	88,827
S16	TX hospice and palliative care nursing	717
S15	TX palliative medicine	14,326
S14	TX Hospice*	26,327
S13	TX "Palliative care"	68,495
S12	S1 OR S2 OR S3 OR S4 OR S5 OR S6 OR S7 OR S8 OR S9 OR S10 OR S11	22,350
S11	TX "Death with Dignity"	615
S10	TX "medical assistance in dying"	79
S9	TX "medical aid in dying"	21
S8	TX "physician assisted suicide"	1528
S7	TX "physician aid in dying"	44
S6	TX "physician assisted dying"	118
S5	TX "aid in dying"	146
S4	TX "voluntary euthanasia"	321
S3	TX euthanasia NOT TX animals	20,927
S2	TX "assisted suicide"	2891
S1	TX "Assisted dying"	533

**Table:** 

S35	S14 AND S19 AND S34	838
S34	S20 OR S21 OR S22 OR S23 OR S24 OR S25 OR S26 OR S27 OR S28 OR S29 OR S30 OR S31 OR S32 OR S33	1,673,440
S33	TX harm*	55,861
S32	TX involv*	270,371
S31	TX improve*	541,477
S30	TX oppose*	10,207
S29	TX embed*	14,367
S28	TX impe*	54,313
S27	TX impact*	307,326
S26	TX cooperation*	11,250
S25	TX collaboration*	76,459
S24	TX rejection*	9734
S23	TX consequence*	69,779
S22	TX compatib*	8952
S21	TX relation*	614,747
S20	TX integrat*	141,327
S19	S15 OR S16 OR S17 OR S18	69,604
S18	TX "hospice and palliative nursing"	4397
S17	TX "palliative medicine"	12,341
S16	TX hospice*	38,817
S15	TX "Palliative Care"	53,210
S14	S3 OR S4 OR S5 OR S6 OR S7 OR S8 OR S9 OR S10 OR S11 OR S12 OR S13	9122
S13	TX "Death with Dignity"	303
S12	TX "medical assistance in dying"	117
S11	TX "medical aid in dying"	39
S10	TX "physician aid in dying"	46
S9	TX "physician assisted suicide"	839
S8	TX "physician assisted dying"	103
S7	TX "aid in dying"	166
S6	TX "voluntary euthanasia"	142
S5	TX "euthanasia" NOT TX animals	7454
S4	TX "assisted suicide"	1749
S3	TX "assisted dying"	539
S2	TX assisted suicide	3609
S1	TX assisted dying	592

## Appendix

MEDLINE Search

CINAHL Search
